# Azithromycin Synergizes with Cationic Antimicrobial Peptides to Exert Bactericidal and Therapeutic Activity Against Highly Multidrug-Resistant Gram-Negative Bacterial Pathogens

**DOI:** 10.1016/j.ebiom.2015.05.021

**Published:** 2015-06-10

**Authors:** Leo Lin, Poochit Nonejuie, Jason Munguia, Andrew Hollands, Joshua Olson, Quang Dam, Monika Kumaraswamy, Heriberto Rivera, Ross Corriden, Manfred Rohde, Mary E. Hensler, Michael D. Burkart, Joe Pogliano, George Sakoulas, Victor Nizet

**Affiliations:** a Department of Pediatrics, University of California, San Diego, La Jolla, CA 92093, USA; b Division of Biological Sciences, University of California, San Diego, La Jolla, CA 92093, USA; c Department of Medicine, University of California, San Diego, La Jolla, CA 92093, USA; d Department of Chemistry and Biochemistry, University of California, San Diego, La Jolla CA 92093, USA; e Central Facility for Microscopy, Helmholtz Centre for Infection Research, 38124 Braunschweig, Germany; f Skaggs School of Pharmacy and Pharmaceutical Sciences, University of California, San Diego, La Jolla, CA 92093, USA; g Rady Children's Hospital, San Diego, CA 92123, USA

**Keywords:** Azithromycin, Macrolides, Antimicrobial peptides, Cathelicidin, LL-37, Antibiotic resistance, Colistin, *Pseudomonas aeruginosa*, *Acinetobacter baumannii*, *Klebsiella pneumoniae*

## Abstract

Antibiotic resistance poses an increasingly grave threat to the public health. Of pressing concern, rapid spread of carbapenem-resistance among multidrug-resistant (MDR) Gram-negative rods (GNR) is associated with few treatment options and high mortality rates. Current antibiotic susceptibility testing guiding patient management is performed in a standardized manner, identifying minimum inhibitory concentrations (MIC) in bacteriologic media, but ignoring host immune factors. Lacking activity in standard MIC testing, azithromycin (AZM), the most commonly prescribed antibiotic in the U.S., is never recommended for MDR GNR infection. Here we report a potent bactericidal action of AZM against MDR carbapenem-resistant isolates of *Pseudomonas aeruginosa*, *Klebsiella pneumoniae*, and *Acinetobacter baumannii*. This pharmaceutical activity is associated with enhanced AZM cell penetration in eukaryotic tissue culture media and striking multi-log-fold synergies with host cathelicidin antimicrobial peptide LL-37 or the last line antibiotic colistin. Finally, AZM monotherapy exerts clear therapeutic effects in murine models of MDR GNR infection. Our results suggest that AZM, currently ignored as a treatment option, could benefit patients with MDR GNR infections, especially in combination with colistin.

## Highlights

• Standard MIC testing conditions overlook a potent activity of azithromycin vs. multidrug-resistant Gram-negative bacteria.
• Colistin and endogenous host defense peptide LL-37 markedly potentiate azithromycin penetration into bacterial cells.
• Azithromycin reduced bacterial load and mortality in mouse models of multidrug-resistant Gram-negative infection.

## 1 Introduction

Hospital-acquired infections, half caused by drug-resistant bacteria (Mauldin et al., 2010), cause ~ 99,000 deaths annually and increase healthcare costs by $5–10 billion in the U.S. alone (Peleg and Hooper, 2010). Recent reports by the U.S. Centers for Disease Control and Prevention (CDC, 2013) and the World Health Organization (WHO, 2014) describe this ever-worsening antibiotic resistance crisis, highlighting the “urgent threat” of emerging carbapenem-resistant Gram-negative rods (GNRs) that forebode the entry of human medicine into a “post-antibiotic era”. Rapid spread of carbapenem resistance in *Pseudomonas aeruginosa* (*PA*), *Klebsiella pneumoniae* (*KP*) and *Acinetobacter baumannii* (*AB*) is of particular concern as effective antibiotic candidates are currently lacking in the development pipeline (Diene and Rolain, 2014).

Unconventional approaches to infectious disease treatment are gaining more attention, including virulence factor inhibition, bacteriophage therapy, probiotics and immune boosting (Cegelski et al., 2008, Hancock et al., 2012). Along these lines, we have probed interactions of conventional antibiotics with antimicrobial effectors of the innate immune system, with encouraging results. Drugs with no direct activity in standard minimum inhibitory concentration (MIC) testing nevertheless sensitized multidrug-resistant (MDR) bacterial pathogens to human antimicrobial peptide killing in vitro, e.g., nafcillin vs. methicillin-resistant *Staphylococcus aureus* (MRSA) or ampicillin vs. vancomycin-resistant *Enterococcus* (VRE), and contributed to clinical resolution in refractory infections (Sakoulas et al., 2012, Sakoulas et al., 2014).

Our recent experiences with β-lactams and MRSA or VRE indicate that simple MIC testing overlooks potential synergies with cationic antibiotics (e.g., daptomycin) and host AMPs (e.g., human cathelicidin LL-37) that promote bactericidal activity in vitro and bacterial clearance in patients (Sakoulas et al., 2012, Sakoulas et al., 2014). We asked whether similar phenomena could be identified in MDR-GNRs to challenge conventional antibiotic treatment paradigms. AZM, the most commonly prescribed antibiotic in the U.S. (51.5 million in 2010) (Hicks et al., 2013), is never recommended for inpatient treatment of serious GNR infections because of poor or absent in vitro activity by standard MIC testing in bacteriologic media. However, antibacterial activity of AZM is enhanced in mammalian tissue culture media vs. standard bacteriologic media (Buyck et al., 2012), a finding reminiscent of observations we made for LL-37 (Dorschner et al., 2006), prompting us to examine its interaction with MDR GNRs more closely.

## 2 Materials and Methods

### 2.1 Bacterial Strains

*P. aeruginosa* (*PA*) strain PA01, *K. pneumoniae* (*KP*) strain K700603, and *A. baumannii* (*AB*) strain AB19606 were obtained from the American Type Culture Collection (ATCC). Human clinical MDR isolates *PA* P4 (lung) and *KP* K1100 (lung) (Fair et al., 2012) were obtained from a tertiary academic hospital in the New York metropolitan area. MDR-*AB* AB5075 (bone) (Zurawski et al., 2012) was obtained from Walter Reed Army Medical Center. All three MDR strains were independently identified and subject to antibiotic susceptibility testing by the clinical microbiology laboratory at the San Diego Veterans Affairs Hospital (table S1). Additional MDR GNR clinical isolates tested were also obtained from a tertiary academic hospital in the New York metropolitan area, except for PA USCD P1, which was obtained from the UC San Diego hospital system. Bacteria were grown overnight in Luria Broth (LB), glycerol was added (30% final), and stocks stored at − 80 °C. Fresh colonies were streaked onto LB plates each week for all experiments.

### 2.2 Antibiotics and Antimicrobial Peptides

For in vitro studies, AZM, colistin sulfate, and ciprofloxacin were purchased from Sigma-Aldrich; erythromycin and clarithromycin were purchased from Fischer Scientific. Stock solutions were prepared in phosphate buffered saline (PBS) at 2560 mg/L for the macrolide antibiotics, 1000 mg/L for colistin, and 10,000 mg/L for ciprofloxacin. Trace amounts of glacial acetic acid were used to prepare AZM, erythromycin and clarithromycin stocks for complete solubility (Barry et al., 2004). LL-37 and TAMRA-tagged LL-37 were purchased from the American Peptide Company; stock solutions were prepared in molecular quality water (Corning Cellgro) at 640 μM and 320 μM, respectively, and stored at − 80 °C. For in vivo studies, AZM for human injection (Sagent Pharmaceuticals) was reconstituted per manufacturer's guidelines (AZM Package Insert, 2013). Pooled human serum was obtained from six healthy consented lab volunteers under a protocol approved by the UCSD Human Research Protection Program and immediately aliquoted and stored at − 80 °C.

### 2.3 Reagents

Mueller–Hinton Broth (MHB, Spectrum Chemicals) was supplemented with CaCl_2_ and MgCl_2_ to make cation-adjusted MHB (Ca-MHB) — final cation concentrations (20–25 mg/L Ca^2 +^ and 10–12.5 mg/L Mg^2 +^). Luria Broth base (LB) was purchased from Hardy Diagnostics. RPMI-1640 was purchased from Invitrogen. Clear phenol free RPMI-1640 used for microscopy studies was purchased from Corning Cellgro. 1-N-phenylnaphthylamine (NPN) was purchased from Sigma-Aldrich and a fresh stock of 500 μM in acetone was made each week.

### 2.4 MIC Determinations

MIC values for AZM, erythromycin, clarithromycin, ciprofloxacin, colistin, and LL-37 were determined using broth microdilution in accordance with the Clinical Laboratory Standards Institute (CLSI) guidelines using Ca-MHB media, the recommended bacteriologic broth, or eukaryotic cell culture media RPMI-1640 supplemented with 5% LB (Sakoulas et al., 2014).

### 2.5 Time-Kill Curves and Serum Survival Assays

Time-kill studies, ± 20% pooled human serum, were performed as previously described (Haste et al., 2011). Bacteria were grown overnight in LB at 37 °C with shaking. Bacterial stocks in PBS were prepared by washing the overnight cultures twice with PBS via centrifugation at 3220 ×*g* at room temperature with a final re-suspension in PBS to an OD_600_ = 0.40. Bacterial stocks in PBS were diluted in Ca-MHB or 5% LB-RPMI to an initial inoculum of 1 × 10^6^ CFU/mL (standard time-kill) or 5 × 10^4^ CFU/mL (serum survival). AZM, erythromycin, clarithromycin, colistin, and LL-37 stocks were diluted in Ca-MHB or 5% LB-RPMI to the assay concentrations indicated. For serum studies, AZM 0.5 mg/L was chosen in order to approximate human plasma concentrations upon intravenous administration of 500 mg of AZM (AZM Package Insert, 2013). Assays were conducted in triplicate in a final volume of 200 μL in 96-well round bottom plates (Costar) ± 20% pooled human serum. The 96-well plates were wrapped in paraffin and placed in a shaking incubator at 37 °C. Aliquots were collected at the indicated times and serially diluted for CFU enumeration; limit of detection = 100 CFU/mL.

### 2.6 Electron Microscopy of Bacterial Gross Morphology

Transmission electron microscopy was performed essentially as described (Sato, 1968). MDR-*AB* was grown overnight in LB at 37 °C with shaking. Bacterial stocks in PBS were prepared by washing the overnight cultures twice with PBS and resuspending in PBS to OD_600_ = 0.40. Then 2.5 mL of each bacterial stock was added to 47.5 mL of Ca-MHB or RPMI + 5% LB media pre-warmed to 37 °C. For treatment, the AZM stock was diluted to a concentration of 0.5 mg/L in the final volume of 50 mL. 50 mL cultures were placed in a shaking incubator at 37 °C for 2 h. Cultures were then centrifuged at 3220 × *g* at room temperature for 10 min. The supernatant was aspirated and bacterial pellets re-suspended in 1 mL of PBS. These 1 mL samples were immersed in modified Karnovsky's fixative (2.5% glutaraldehyde and 2% paraformaldehyde in 0.15 M sodium cacodylate buffer, pH 7.4) for at least 4 h, post-fixed in 1% osmium tetroxide in 0.15 M cacodylate buffer for 1 h, and stained en bloc in 2% uranyl acetate for 1 h. Samples were dehydrated in ethanol, embedded in Durcupan epoxy resin (Sigma-Aldrich), sectioned at 50–60 nm on a Leica UCT ultramicrotome, and picked up on Formvar and carbon-coated copper grids. Sections were stained with 2% uranyl acetate for 5 min and Sato's lead stain for 1 min. Grids were viewed using a Tecnai G2 Spirit BioTWIN transmission electron microscope and photographs were taken with an Eagle 4 k HS digital camera (FEI). Images were taken from multiple random fields at 1200 ×, 2900 ×, 23,000 ×; and gross morphology was analyzed in a blinded fashion.

### 2.7 Fluorescence Microscopy for MDR-AB Cytological Profiling

The fluorescence microscopy studies for MDR-*AB* were performed as previously described (Nonejuie et al., 2013). These microscopy studies required higher concentrations of AZM because the concentration of MDR-*AB* used was 100 × higher (5 × 10^7^ CFU/mL) compared to the concentration used in the MIC assays (5 × 10^5^ CFU/mL). All AZM concentrations used in these studies are pharmacologically obtainable in human tissue. Single MDR-*AB* colonies were picked from LB plates and grown in LB or RPMI + 5% LB overnight. Overnight cultures were then diluted 1:100 into fresh Ca-MHB or RPMI + 5% LB media. When an OD_600_ = 0.20 was reached, antibiotics were added to exponentially growing bacteria. Cultures were placed on a shaker at 30 °C and collected after 1 h or 2 h and stained with 1 μg/mL FM4-64 2 μg/mL DAPI, and 0.5 μM SYTOX-Green (Molecular Probes/Invitrogen). In the case of NBD-tagged AZM, SYTOX-Green dye was omitted and cell cultures were washed with fresh media before staining. Stained cultures were centrifuged at 300 × *g* for 30 s in a microcentrifuge and resuspended in approximately 5% original volume. 3 μL of concentrated cells were transferred onto a pad containing 1.2% agarose and 20% LB medium for microscopy. The exposure time of each excitation was the same for each experimental replicate included in the statistical analysis of all treatments. All images were analyzed using ImageJ software v1.48f and CellProfiler 2.0. Fluorescence intensity analysis was performed on non-deconvolved images. Average DAPI, SYTOX Green, or NBD intensity per cell was measured and respective background intensities subtracted. Finally, the intensity of treated cells was normalized by the intensity of untreated cells from the same experiment set, making intensity data from different experimental sets comparable as relative intensity. Protein translation inhibition phenotype was automatically calculated by the degree of DNA condensation defined by the ratio of DNA area over total cell membrane area. Cells with a ratio less than half of those calculated from untreated control cells were considered to have condensed toroid nucleoids (Nonejuie et al., 2013). Fluorescence intensity and protein translation inhibition population data were obtained by counting > 500 cells from random microscopy fields per condition per experimental replicate, with 3 independent replicates. The fluorescence microscopy for LL-37 cell binding was performed in a similar manner except that after the MDR-*AB* cultures were incubated in 0.5 mg/L of AZM versus control for 2 h, 2 μM of TAMRA-tagged LL-37 was added and the cultures incubated for an additional 30 min before staining and analysis.

### 2.8 Mouse AZM Dosing

Standard human dosing of AZM is 500 or 1000 mg administered orally or intravenously q 24 h (7.7 or 15 mg/kg for an average adult). Mice metabolize AZM 29 times faster than humans (Hoffmann et al., 2007), and 50 mg/kg AZM given subcutaneously to mice approximates 500 mg given intravenously to human patients (Beigelman et al., 2009). We therefore chose to administer 50 or 100 mg/kg AZM subcutaneously in our murine infection models once every 24 h.

### 2.9 Mouse Pneumonia Models

The murine pneumonia mode was performed with slight modifications as previously described (Revelli et al., 2012). All intratracheal infections were performed in a blinded fashion with respect to AZM or PBS treatment. For the MDR-*AB* lung infection model, 8-wk-old female C57Bl/6J mice (Jackson Labs) were used, divided randomly into matched treatment and control groups. MDR-*AB* cultures were grown overnight in LB at 37 °C with shaking and then re-grown in the morning in fresh LB to a concentration of OD_600_ = 0.40. Bacteria were washed twice with PBS via centrifugation at 3220 ×*g* at room temperature and concentrated in PBS to yield 5 × 10^6^ CFU in the 30 μL, the inoculation volume. Mice were anesthetized with 100 mg/kg ketamine and 10 mg/kg xylazine. Once sedated, the vocal chords were visualized using an operating otoscope (Welch Allyn) and 30 μL of bacteria or PBS was instilled into the trachea during inspiration using a plastic gel loading pipette tip. Mice were placed on a warmed pad for recovery and given one subcutaneous dose of 50 mg/kg or 100 mg/kg human AZM for injection reconstituted in PBS. Mice were sacrificed with CO_2_ for bacterial counts or for analysis of their broncho alveolar fluid (BALF) 24 h after infection. To enumerate total surviving bacteria in the lungs, both lung lobes were removed and placed in a 2 mL sterile micro tube (Sarstedt) containing 1 mL of PBS and 1 mm silica beads (Biospec). Lungs were homogenized by shaking twice at 6000 rpm for 1 min using a MagNA Lyser (Roche), with the specimens placed on ice as soon as they were harvested. Aliquots from each tube were serially diluted for CFU enumeration on LB plates. For BALF collection and analysis, an incision was made in the trachea and the lungs slowly inflated with 700 μL of chilled PBS using a 1 mL syringe topped with a 20G blunt needle tip (Harvard Apparatus). The PBS was slowly withdrawn, and the recovered BALF was spun at 1200 ×*g* at 4 °C for 10 min. The supernatant (350 μL) was analyzed for the mouse pro-inflammatory cytokines mIL-1β, mIL-6, and mMIP-2 (R&D Systems ELISA Kits). Cell pellets were resuspended in chilled PBS to a final volume of 500 μL. Samples were enumerated with a hemocytometer for total leukocyte number. 100 μL was spun onto glass microscopy slides using a Shandon Cytospin 3 (Thermo Scientific). Dry glass slides were stained with Wright–Giemsa stain, and > 200 leukocytes were counted per animal using a light microscope for neutrophil and alveolar macrophage enumeration. For the MDR-*AB* lung infection survival experiment 5 × 10^7^ CFU were given in 36 μL of PBS. One dose of 100 mg/kg AZM or 100 μL of PBS control was given subcutaneously immediately after the infection (time 0) and a second dose was given 24 h later. The MDR *KP* and *PA* intra-tracheal murine lung infections were performed in a similar manner except that 8-week-old female CD1 mice (Charles River Labs) were used and the animals were sacrificed 36 h after infection for enumeration of surviving bacteria. The inocula for MDR-*KP* and MDR-PA infection were 1.5 × 10^7^ CFU and 1 × 10^7^ CFU in 40 μL of PBS, respectively. The *AB* foreign body infection studies were performed as previously described (Kadurugamuwa et al., 2003). Outcome assessment was not blinded to AZM vs. PBS groups, but these involved objective (e.g., mortality, bacterial counts, cytokine levels) not subjective data. All animal studies were performed under protocols approved by the UCSD Institutional Animal Use and Care Committee.

### 2.10 Statistical Analysis

All statistics were performed using GraphPad Prism version 5.0 2-way ANOVA, 1-way ANOVA, two tailed Student's t-test, and log-rank test were performed as described in each figure legend.

## 3 Results

### 3.1 AZM is Highly Bactericidal vs. MDR GNRs in Tissue Culture Media

**Fig. 1:**
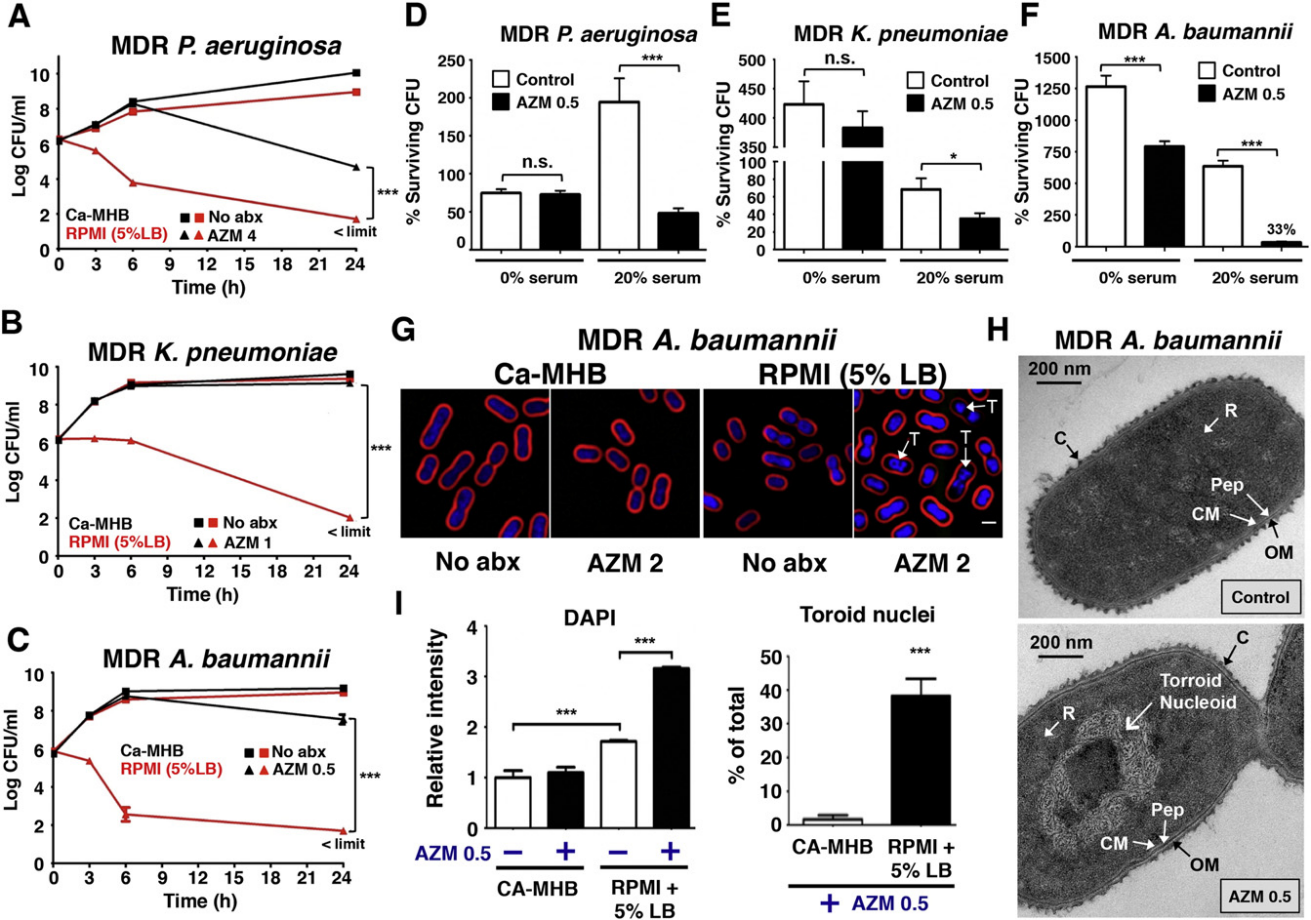
AZM bactericidal activity against GNRs in eukaryotic media and human serum. AZM concentrations = mg/L. (A–C) Time-kill curves demonstrating AZM MBC against MDR *PA*, *KP*, and *AB* in eukaryotic media (RPMI + 5% LB) vs. bacteriologic broth (Ca-MHB). Mean of triplicates from 3 independent experiments ± SEM ***P < 0.001; two-way ANOVA; limit of detection 50 CFU/mL. (D–F) 5 × 10^4^ CFU/mL bacteria incubated at 37 °C for 2 h in RPMI alone (0% serum), AZM 0.5, 20% serum, and AZM 0.5 + 20% serum. Data show % viable CFU vs. initial inoculum; mean of triplicates from 3 independent experiments ± SEM. *P < 0.05, ***P < 0.001; two-tailed Student's t-test. (G) MDR *AB* (5 × 10^7^ CFU/mL) grown in Ca-MHB vs. RPMI + 5% LB were treated for 2 h with AZM 2 and stained for fluorescence microscopy: FM4-64 (red cell membrane stain), DAPI (blue DNA stain), “T” denotes toroid shaped nucleoid. For ready visualization, the concentration of MDR *AB* was 100-fold higher than in MIC assays; thus a higher concentration AZM was used. (H) Transmission electron microscopy images representative of 2 independent experiments with logarithmic growth phase MDR *AB* treated for 2 h with AZM 0.5; C = Capsule; CM = Cell Membrane; Pep = Peptidoglycan; OM = Outer Membrane; R = Ribosomes. (I) Bar graphs generated from unbiased software analysis of multiple random microscopy fields with > 500 cells counted per condition per experimental replicate. Data representative of 3 independent experiments plotted as mean ± SEM; additional microscopy details in Materials and methods section. ***P < 0.001; one-way ANOVA and two tailed Student's t-test.

For each opportunistic GNR pathogen (*PA*, *KP* and *AB*) we tested a common model strain plus a corresponding extremely MDR human isolate (Supplementary Table 1). AZM MICs were determined by Clinical and Laboratory Standard Institute (CLSI) broth microdilution methodology (CLSI, 2008) using either the recommended cation-adjusted Mueller–Hinton broth (Ca-MHB) or eukaryotic cell culture media RPMI-1640 supplemented with 5% Luria broth (RPMI + 5% LB) (Supplementary Table 2). In every case, a ≥ 30-fold reduction in MIC was seen in RPMI + 5% LB vs. Ca-MHB, rendering all organisms susceptible by the accepted breakpoint of ≤ 8 mg/L for *Campylobacter* and *S. aureus*. In contrast, no marked changes in MIC of fluoroquinolone ciprofloxacin were observed for model strains (all sensitive) or MDR clinical isolates (all resistant) in RPMI + 5% LB vs. Ca-MHB (Supplementary Table 2). A similarly profound reduction in MIC in RPMI + 5% LB vs. Ca-MHB was also observed for an expanded panel of 11 additional MDR GNR clinical isolates (Supplementary Table 3). Two additional macrolides, erythromycin and clarithromycin, also demonstrated dramatically higher activity against MDR GNRs in RPMI + 5% LB versus Ca-MHB, although AZM was the most potent of the macrolides tested (Supplementary Table 2). Time-kill curves determined the mean bactericidal concentration (MBC, reduction in CFU by 2log_10_) of AZM against the MDR-GNRs in RPMI + 5% LB media (Fig. 1A–C). With an initial inoculum of 1 × 10^6^ CFU/mL, the MBC of AZM against MDR-*PA*, -*KP* and -*AB* were 4 mg/L (MIC = 2 mg/L), 1 mg/L (MIC = 1 mg/L) and 0.5 mg/L (MIC = 0.5 mg/L) respectively. While all three MDR-GNRs achieved rapid logarithmic growth in both RPMI + 5% LB and Ca-MHB, AZM was markedly more bactericidal in the eukaryotic tissue culture media (Fig. 1A–C): a striking 6-logfold increased killing for *KP* and *AB* and 2-logfold increased killing for *PA*. For MDR-*PA* and -*AB*, AZM 4 mg/L (< 1/16 MIC) resulted in significant killing even in Ca-MHB once bacteria reached stationary growth phase (Fig. 1A and C), consistent with reported AZM bactericidal activity against stationary phase antibiotic-sensitive *PA* (Imamura et al., 2005). AZM activity against all three MDR pathogens was further enhanced in the presence of 20% human serum (Fig. 1D–F), mirroring a prior observation of serum potentiation of AZM activity against *Escherichia coli* and *S. aureus* (Pruul and McDonald, 1992). Serial passage of all three MDR pathogens for 10 consecutive days at sub-minimum inhibitory concentrations of AZM in RPMI + 5% LB media demonstrated no increase in resistance to AZM over this time frame (Fig. S1).

### 3.2 Enhanced AZM Permeability in Tissue Culture Media Triggers Nucleoid Collapse

AZM binds the 50S large ribosomal subunit at the polypeptide exit tunnel, blocking protein synthesis (Kannan et al., 2012). We employed fluorescence microscopy-based bacterial cytological profiling (BCP) (Nonejuie et al., 2013) of MDR-*AB* to identify the cellular pathway perturbed by AZM treatment in RPMI + 5% LB media but not Ca-MHB. BCP revealed that nucleoid collapse into a low energy toroid shape, a hallmark of protein synthesis inhibition (Nonejuie et al., 2013, Zusman et al., 1973), was markedly increased in *AB* treated with AZM in RPMI + 5% LB (Fig. 1G), a finding corroborated by transmission electron microscopy of treated cells (Fig. 1H). Increased staining intensity of the DNA dye DAPI in RPMI + 5% LB vs. Ca-MHB even without AZM suggested increased membrane permeability in the cell culture media (Fig. 1I). DAPI staining of MDR-*AB* was further increased upon AZM treatment, possibly reflecting impaired efflux pump function seen in antibiotic-sensitive *PA* treated with AZM in RPMI (Buyck et al., 2012).

### 3.3 Colistin Permeabilization Markedly Potentiates AZM Bactericidal Activity vs. MDR GNRs

**Fig. 2:**
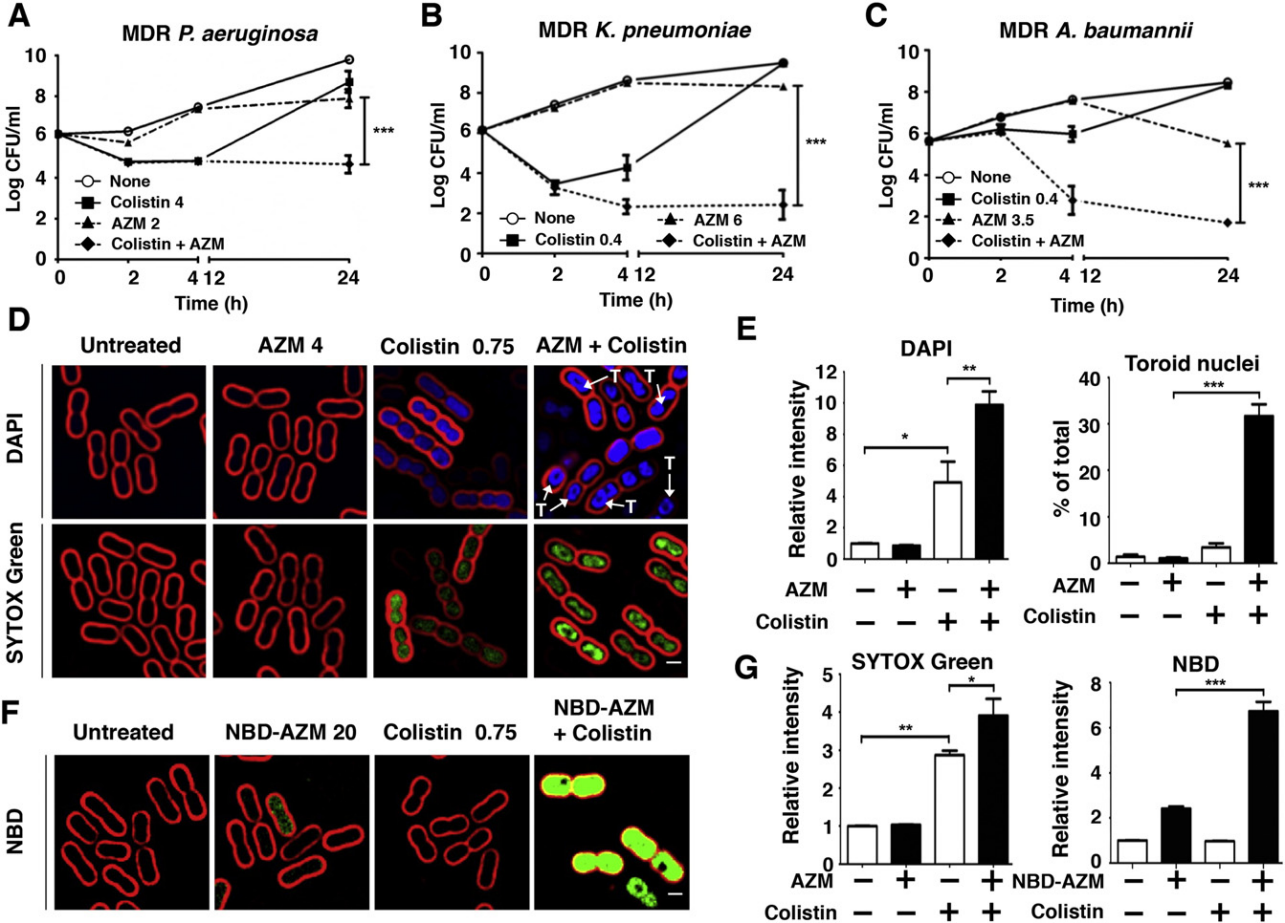
Colistin synergizes with AZM by increasing GNR outer membrane permeability. All assays conducted using the bacteriologic media Ca-MHB. (A–C) Time-kill curves demonstrate the effect of AZM, colistin, or both in combination against MDR *PA*, *KP* and *AB*. Data plotted are mean ± SEM and represent the average of triplicates from 3 independent experiments. ***P < 0.001; two-way ANOVA. (D and F) Growth phase MDR *AB* (5 × 10^7^ CFU/mL) treated for 1 h with AZM (unlabeled or NBD-tagged), colistin, or a combination of both, then stained for fluorescence microscopy: FM4-64 (red cell membrane stain), DAPI (blue DNA stain), SYTOX Green (green DNA stain), and NBD-AZM (green NBD-tagged AZM), “T” = toroid shaped nucleoid. DAPI blue DNA stain present in “Untreated” and “AZM 4” panels. Since signal from all conditions was normalized to untreated controls and “AZM + Colistin” treated bacteria had 10-fold higher DAPI signal intensity, blue signal was reduced equally across all four displayed DAPI panels to prevent washout and enable visualization of the toroid structures in the cells with high DAPI signal intensity. (E and G) Bar graphs were generated from unbiased software analysis of multiple random microscopy fields with > 500 cells counted per condition per experimental replicate. Data representative of 3 independent experiments and plotted as the mean ± SEM — additional microscopy details in methods section. *P < 0.05, **P < 0.01, ***P < 0.001; one-way ANOVA.

Colistin is an antibiotic of last resort for carbapenem-resistant GNR infections (Biswas et al., 2012), but its use is complicated by marked dose-dependent nephrotoxicity and uncertainties in optimal dosing (Spapen et al., 2011). We asked whether AZM could act synergistically with colistin, even in standard Ca-MHB media in which AZM alone has little or no activity. At a sub-MIC (Supplementary Table 2) and pharmacologically attainable dose of each drug, marked synergy (Leonard, 2012) of AZM + colistin was observed against MDR-*PA* (additional reduction in CFU by 2log_10_), -*KP* (by 5log_10_) and -*AB* (by 3log_10_) (Fig. 2A–C). BCP showed strongly increased DAPI staining and toroid nucleoid morphology in MDR-*AB* treated with AZM + colistin vs. either agent alone (Fig. 2D and E). A membrane permeability effect was corroborated as bacterial cell entry of the fluorescent dye SYTOX green (MW = 600 Da vs. 749 Da for AZM) was increased 3-fold by colistin and 4-fold by colistin + AZM (Fig. 2D and G). Indeed, colistin markedly enhanced entry of fluorescently tagged AZM (NBD-AZM (Matijasic et al., 2012)) into MDR-*AB* cells (Fig. 2F and G). A cationic peptide antibiotic, colistin permeabilizes the bacterial cell envelope (Koike et al., 1969), and we surmise it facilitates AZM entry whereupon the latter drug can exert its classical ribosomal protein synthesis activity. Erythromycin and clarithromycin also showed significant synergy with colistin (Fig. S2), with azithromycin the most potent of the three macrolides tested.

### 3.4 Pronounced Bactericidal Synergy of AZM with Human Cathelicidin LL-37 vs. MDR GNRs

**Fig. 3:**
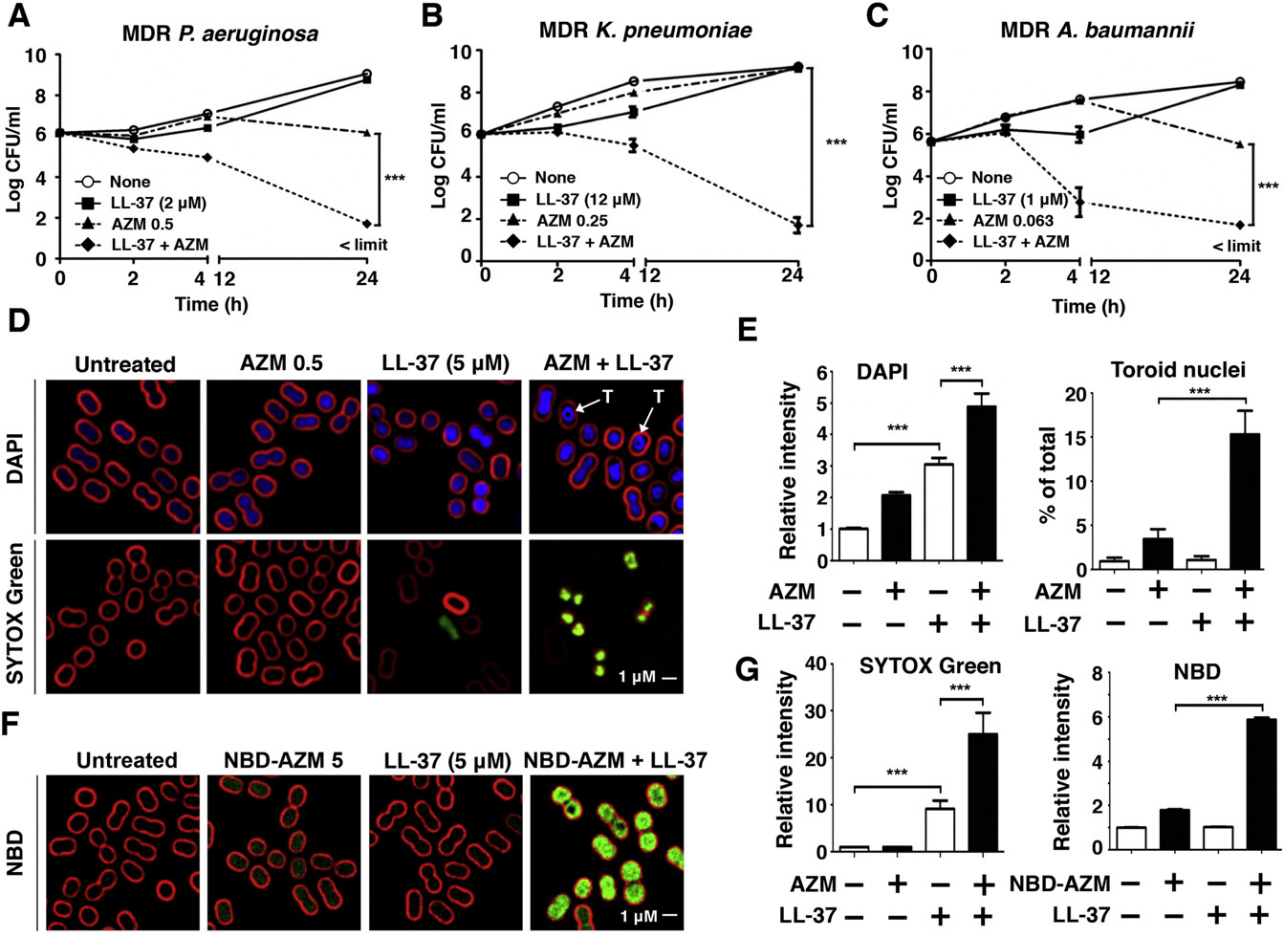
Cationic AMP LL-37 synergizes with AZM by increasing the outer membrane permeability of MDR-GNRs. All assays conducted using the eukaryotic media RPMI + 5% LB. (A–C) Time-kill curves demonstrating effects of AZM, LL-37, or both in combination against MDR *PA*, *KP*, and *AB*. Data plotted are mean ± SEM and represent the average of triplicates from 3 independent experiments. ***P < 0.001; two-way ANOVA. Limit of detection = 50 CFU/mL (D and F) Logarithmic growth phase MDR *AB* (5 × 10^7^ CFU/mL) were treated for 2 h with AZM (unlabeled or NBD-tagged), LL-37, or a combination of both, then stained for fluorescence microscopy: FM4-64 (red cell membrane stain), DAPI (blue DNA stain), SYTOX Green (green DNA stain), and NBD-AZM (green NBD-tagged AZM), “T” denotes toroid shaped nucleoid. (E and G) Bar graphs generated from unbiased software analysis of multiple random microscopy fields with > 500 cells counted per condition per experimental replicate. Data representative of 3 independent experiments and plotted as the mean ± SEM. — additional microscopy details in methods section. ***P < 0.001; one-way ANOVA.

Endogenous cationic AMPs are critical to mammalian innate immunity against invasive bacterial infection (Gallo and Hooper, 2012). Given the observed synergy of AZM with the bacterial-derived AMP colistin, we hypothesized that AZM could be potentiated by LL-37, a cationic AMP produced abundantly by phagocytic and epithelial cells during infection (Cederlund et al., 2011). At sub-MIC doses (Supplementary Table 2), marked synergy of AZM + LL-37 was observed against MDR-*PA* (additional reduction in CFU by 4log_10_), *KP* (7log_10_) and *AB* (4log_10_) (Fig. 3A–C). For MDR-*AB*, AZM 0.0625 mg/L (1/8th MIC) + 1 μM LL-37 (1/4th MIC) resulted in > 99% killing within 4 h. BCP showed increased DAPI staining and toroid morphology of nucleoids in MDR-*AB* treated with AZM + LL-37 vs. either agent alone (Fig. 3D and E). SYTOX green entry into the bacterial cell was increased 3-fold in the presence of LL-37 and 5-fold in the presence of LL-37 + AZM (Fig. 3D and G). LL-37 also markedly enhanced entry of NBD-AZM into MDR-*AB* cells (Fig. 3 F and G), consistent with the known activity of LL-37 to interfere with GNR cell wall biogenesis and cytoplasmic membrane integrity (Sochacki et al., 2011). Studies with 1-N-phenylnaphthylamine (NPN), a validated marker for outer membrane permeability of GNRs (Helander and Mattila-Sandholm, 2000), verified that sub-MIC concentrations of colistin or LL-37 increased outer membrane permeability of the MDR-*AB* strain (Fig. S3). Interestingly, AZM pretreatment of MDR-*AB* significantly enhanced binding of TAMRA-tagged LL-37 to the bacterial outer membrane, suggesting bidirectional synergy (Fig. S4).

### 3.5 AZM Monotherapy Promotes Clearance of MDR GNR in Murine Infection Models

**Fig. 4:**
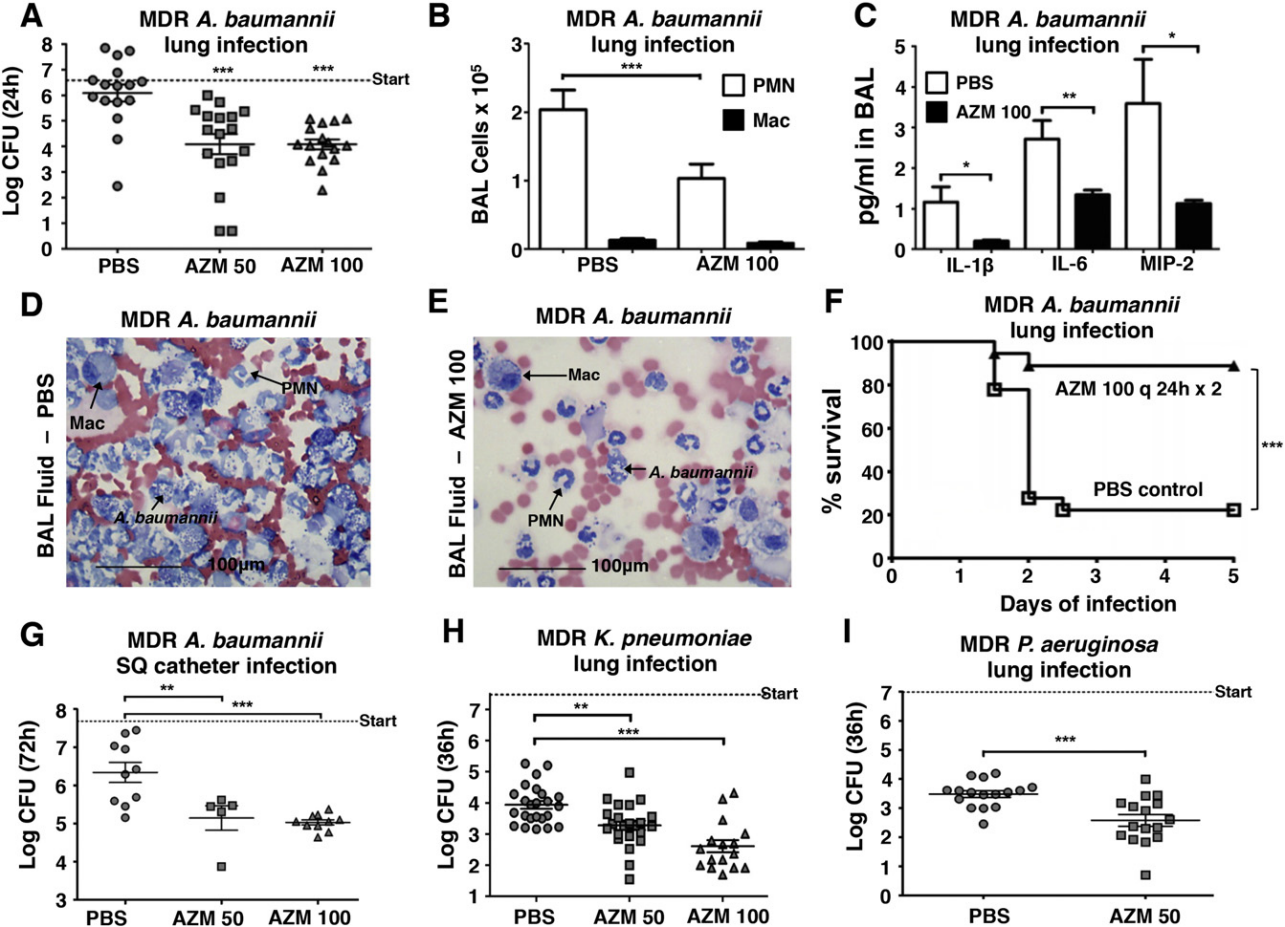
AZM activity as monotherapy against MDR-GNRs in vivo. (A) C57BL/6J mice were infected intratracheally (i.t.) with MDR *AB*. Lungs homogenized at 24 h; n = 16 for PBS control, 17 for AZM 50 mg/kg, and 16 for AZM 100 mg/kg. (B) Wright–Giemsa stained BALF from C57BL/6J mice infected i.t. with *AB*. Total neutrophils and macrophages enumerated by light microscopy + hemocytometer counts; n = 19 PBS control and 20 AZM 100 mg/kg-treated mice. PMN = polymorphonuclear leukocyte/neutrophil, Mac = macrophage. (C) ELISA detection of inflammatory cytokines in the BALF of mice from (b). (D and E) Light microscopy of Wright–Giemsa stained BALF of mice from (b); Mac = alveolar macrophage; PMN = neutrophil. (F) Survival of C57BL/6J mice infected i.t. with 6 × 10^7^ CFU of *AB*. Mice received two total doses of AZM versus PBS spaced 24 h apart. n = 18 PBS control and 18AZM 100 mg/kg-treated mice. (G) 1 cm catheter fragment coated with *AB* was implanted subcutaneously into CD-1 mice; results of 2 independent experiments. N = 10 PBS; 5 AZM 50 mg/kg-treated, 10 Az 100 mg/kg-treated mice (H) CD-1 mice infected i.t. with MDR *KP*. Lungs homogenized at 36 h. N = 24 PBS control, 23 AZM 50 mg/kg-treated, and 16 AZM 100 mg/kg-treated mice (i) CD-1 mice infected i.t. with MDR *PA*. Lungs homogenized at 36 h; results of 2 independent experiments. n = 16 PBS control and 16 AZM 50 mg/kg-treated mice. Data plotted as mean ± S.E.M. of 3 independent experiments unless otherwise stated. For in vivo mouse studies, AZM was dosed subcutaneously once every 24 h. Dashed line “start” denotes the initial inoculum. *P < 0.05 **P < 0.01 ***P < 0.001; one-way ANOVA in vivo studies, log-rank test for survival, two-tailed Student's t test in vitro studies.

Activity in mammalian culture media and synergy with host AMPs suggested AZM could be effective against MDR-GNR pathogens in vivo. AZM concentrates in soft tissues and phagocytes (Amsden, 2001, Girard et al., 1990), pharmacodynamic features providing high drug levels at infectious foci where endogenous cationic AMPs are deployed. We studied an intratracheal infection model of MDR-*AB* pneumonia with AZM as the sole therapeutic agent. A single subcutaneous dose of 50 or 100 mg/kg AZM was chosen to achieve serum concentrations mimicking those found with typical 500 or 1000 mg AZM intravenous dosing given to human patients (Beigelman et al., 2009). A single AZM dose reduced by 2log_10_ or 99% the amount of MDR *AB* recovered from lungs 24 h after challenge (Fig. 4A). Examination of bronchial alveolar lavage fluid (BALF) revealed that AZM-treated animals had 50% fewer neutrophils (alveolar macrophages unchanged) (Fig. 4B), significantly reduced pro-inflammatory cytokines IL-1β, IL-6, and MIP-2 (Fig. 4C), and histological evidence of reduced neutrophil and bacterial infiltrations (Fig. 4D and E). Increasing the inoculum of MDR-*AB* 10-fold produced significant mortality within 48 h. One dose of 100 mg/kg AZM after initial infection, followed by a second dose at 24 h, improved 5d survival from 22% to 89% (Fig. 4F). In a subcutaneous foreign body infection model, daily AZM reduced by > 90% the quantity of MDR-*AB* recovered after 3d (Fig. 4G). Reductions of up to 10-fold in bacterial counts were also observed for AZM monotherapy in both MDR-*KP* and -*PA* lung infection models (Fig. 4H and I). Low dose colistin therapy alone did not lead to a significant reduction in the amount of MDR *AB* recovered from lungs 24 h after challenge. However, when a low dose of AZM was combined with a low dose of colistin, a significant reduction in lung bacterial CFU was achieved (Fig. S5).

## 4 Discussion

The continual emergence and rapid spread of MDR-GNRs in hospitals around the world has alarmed physicians, public health epidemiologists and government agencies, spurring urgent calls to action (World Health Organization, 2014, Centers for Disease Control and Prevention, 2013). Due to historical precedent and the intrinsic appeal of a “gold standard”, a single bioassay, the MIC performed in bacteriological media, has come to dominate evaluation of antibiotic efficacy, from the earliest stages of the drug development process, to management of patients based on clinical isolate testing, to establishment of hospital formularies. However, even before the first diagnostic encounter with a physician, a patient's infection is already being combatted by numerous endogenous antimicrobial components, including cationic AMPs of the innate immune system. We believe there is value in analyzing the action of pharmaceutical antibiotics in the richer context of these host defense factors.

Using traditional susceptibility testing methods, the familiar antibiotic AZM shows negligible activity against MDR-GNRs. A small number of prior studies examining multiple combinations of antibiotics in vitro have provided clues that AZM can exhibit activity against GNRs (Appleman et al., 2000, Timurkaynak et al., 2006) or synergize with a polymyxin type antibiotic (Vaara et al., 2010), but without mechanistic or in vivo analysis or suggestions of clinical application. To this day, AZM remains excluded from the antibiotic testing panel reported to physicians when such bacteria are recovered from the blood, sputum or urine of hospitalized patients. Here we show that AZM has potent bactericidal activity against representative strains of the most fearsome MDR-GNRs when tested in eukaryotic cell media (+/− human serum) and in vivo murine models of infection. AZM entry and activity against MDR-GNRs is synergistically enhanced when the bacterial outer membrane is perturbed by cationic human AMP LL-37 or by the last-line antibiotic colistin. Because mutation of the *oprM* efflux pump system in *P. aeruginosa* has been associated with increased AZM sensitivity, and protein synthesis inhibition by AZM can reduce oprM gene expression (Buyck et al., 2012), the enhanced entry of AZM in eukaryotic media and in synergy with colistin or LL-37 that we have demonstrated may initiate a positive feedback loop to increase effective intracellular levels of the antibiotic.

Of note, AZM is touted to have anti-inflammatory effects in lung tissues during infection (Yamada et al., 2013), and several studies of short- or long-term AZM administration in patients with cystic fibrosis, a disease characterized by chronic recurrent pulmonary infection with *PA* and other MDR-GNR bacterial pathogens, have shown reductions in exacerbations and improvement in respiratory capacity (reviewed in Zarogoulidis et al., 2012). Our findings raise the possibility that these benefits could reflect, at least in part, a direct and otherwise unanticipated bactericidal activity of the drug. The macrolides, and azithromycin in particular, have a number of reported non-bactericidal properties that could further complement the in vivo efficacy we observed in our murine models of infection. For example, against various Gram-negative organisms, AZM has been shown to impair bacterial biofilms (Gillis and Iglewski, 2004, Molinari et al., 1993), virulence factor production (Molinari et al., 1993), motility (Molinari et al., 1992), quorum sensing (Hoffmann et al., 2007), and adherence to host epithelial cells (Baumann et al., 2001, Braga and Piatti, 1993).

The major limitation of this study was the use of murine models of MDR GNR infection as opposed to human clinical cases. Laboratory mice are relatively resistant to infection by human clinical isolates of MDR GNRs requiring high inocula to create a disease phenotype. Furthermore, young mice with normal immunity were tested, whereas many human patients that develop MDR GNR infection have multiple co-morbidities including weakened immunity (e.g., from cancer chemotherapy) or dependence on a medical device (e.g., mechanical ventilator). Future randomized clinical trials will be needed to validate whether or not AZM has therapeutic efficacy in older and sicker human patients suffering from MDR GNR infections.

In summary, our studies provide an experimental rationale to further explore AZM as adjunctive therapy in MDR-GNR infections. In particular, we have demonstrated for the first time that colistin or LL-37 permeabilization of Gram-negative outer membranes facilitates entry of the large AZM molecule. Potentially, additional antibiotics, currently disregarded for various clinical indications due to poor penetration into Gram-negative bacterial membranes in standard MIC testing, may likewise have unrecognized in vivo activities when host immune factors perturb bacterial membrane integrity. Most immediately, the AZM synergy with colistin we demonstrate here may allow lower dose, colistin-sparing regimens that reduce adverse drug effects. Continued examination of pharmacodynamic interactions between administered antibiotics and endogenous AMPs of the innate immune system may reveal novel treatment strategies for challenging infections.

## Declaration of Interests

The authors have no competing interests to declare.

## Author Contributions

L.L., P.N., J.M., A.H., J.O., J.P., G.S. and V.N. planned and designed the experiments. L.L., P.N., J.M., A.H., J.O., Q.D., M.K., R.C., and M.E.H. conducted the experiments. M.R. interpreted the electron microscopy studies. H.R. and M.D.B. improved the published synthesis and product analysis of NBD-AZM. L.L., P.N., J.M., A.H., J.O., M.E.H., J.P., G.S. and V.N. interpreted data. L.L. and V.N. drafted and revised the manuscript.

## Funding

This research was supported in part by the National Institute of Health grants HD071600 (to G.S. and V.N.), AI057153, AI052453, AR052728 (to V.N.) and GM073898 (to J.P.). L.L. was supported by the UCSD Medical Scientist Training Program (T32 GM007198), J.M. by the UCSD Training Program in Cellular and Molecular Pharmacology (T32 GM007752) and M.K. by the UCSD Infectious Diseases Fellowship Training Program (T32 AI007036). The funding agency has no role in the actual experimental design, analysis, or writing of this manuscript.
