# Supplementary material for: Azithromycin Synergizes with Cationic Antimicrobial Peptides to Exert Bactericidal and Therapeutic Activity Against Highly Multidrug-Resistant Gram-Negative Bacterial Pathogens
Source: eBioMedicine. 2015 Jun 10;2(7):690–8. doi: 10.1016/j.ebiom.2015.05.021 (PMC4534682; doi:10.1016/j.ebiom.2015.05.021)
Supplement: Supplementary file 1 — Supplementary material 1. [file mmc1.docx]

**RESEARCH IN CONTEXT**

Potentially life-threatening infections with multidrug-resistant Gram-negative rod (MDR-GNR) bacteria increasingly threaten modern medicine. By standard assays, the common and safe antibiotic azithromycin is considered ineffective against MDR-GNR infections, and therefore never recommended. We show that under conditions more similar to normal human body fluids, and in synergy with normal components of the human innate immune system, azithromycin can enter GNR bacterial cells and efficiently kill them. Strikingly, azithromycin effectively treated MDR‑GNR infections in mice. In the current era of increasing antibiotic resistance, a more holistic approach to antibiotic evaluation, incorporating the host innate immune system, may provide new therapeutic options.
