# Supplementary material for: Azithromycin Synergizes with Cationic Antimicrobial Peptides to Exert Bactericidal and Therapeutic Activity Against Highly Multidrug-Resistant Gram-Negative Bacterial Pathogens
Source: eBioMedicine. 2015 Jun 10;2(7):690–8. doi: 10.1016/j.ebiom.2015.05.021 (PMC4534682; doi:10.1016/j.ebiom.2015.05.021)
Supplement: Supplementary file 2 — Supplementary material 2. [file mmc2.pdf]

- Supplementary Materials -

Azithromycin Synergizes with Cationic Antimicrobial Peptides to Exert Bactericidal and Therapeutic Activity Against Highly Multidrug-Resistant Gram-Negative Bacterial Pathogens

Leo Lin[a*], Poochit Nonejuie[b†], Jason Munguia[a†], Andrew Hollands[a], Joshua Olson[a], Quang Dam[a], Monika Kumaraswamy[a], Heriberto Rivera[c], Ross Corriden[a], Manfred Rohde[d], Mary E. Hensler[a], Michael D. Burkart[c], Joe Pogliano[b], George Sakoulas[a], Victor Nizet[a,e,f*]

Supplementary Materials and Methods

Supplementary Figures

Fig. S1. Serial passage for 10 days of MDR GNR isolates at sub-minimum inhibitory concentrations of azithromycin to test for resistance evolution.

Fig. S2. Colistin synergizes with azithromycin, erythromycin, and clarithromycin to kill MDR GNR pathogens *in vitro*.

Fig. S3. Treating MDR *Acinetobacter baumannii* with sub-MIC LL-37 or colistin significantly increases outer membrane permeability to 1-N-phenylnaphthylamine (NPN).

Fig. S4. Pre-treating MDR *Acinetobacter baumannii* with azithromycin increases outer membrane binding of LL-37.

Fig. S5. Addition of low dose azithromycin to reduced dose colistin in treatment of MDR GNRs *in vivo*.

Fig. S6. Confirmation of structure and purity of synthesized NBD-tagged AZM.

Supplementary Tables

Table S1. Three multidrug (including carbapenem)-resistant Gram-negative bacterial strains examined in this study.

Table S2. Minimum inhibitory concentration (MIC) of Gram-negative bacterial strains used in this study to azithromycin, ciprofloxacin, erythromycin, clarithromycin, colistin and human cathelicidin LL-37.

Table S3. Minimum inhibitory concentration (MIC) of azithromycin vs. a panel of contemporary clinical isolates of multidrug-resistant Gram-negative bacteria.

Supplementary Materials and Methods

Serial Passage of MDR GNR isolates. Cultures of *Pseudomonas aeruginosa*, strain P4, *Klebsiella pneumoniae*, strain K1100, and *Acinetobacter baumannii*, strain AB5075 were grown at 1/2 and 1/4 their AZM MIC in 5ml of RPMI + 5%LB. Cultures were placed in a 37^{0}C shaker, and 24 η later, the highest concentration of AZM in which dense bacterial growth was observed was recorded. 50μl of this dense culture growth was then used to inoculate three new tubes containing 5ml of fresh media and 2, 1, and 1/2 the original concentration of AZM respectively. Serial passage was performed twice and data represent one of two independent experiments.

NPN bacterial outer membrane permeability assay. 1-N-phenylnaphthylamine (NPN) is a molecule that becomes fluorescent when it comes into contact with the inner membrane of Gram-negative bacteria, and this property was used to study MDR-*AB* membrane permeability {Helander, 2000 #25}. Overnight cultures of MDR-*AB* grown in LB at 37°C with shaking were washed twice with PBS via centrifugation at 3220x*g* at room temperature and re-suspended to an OD_{600}=0.40 in 8ml of Ca-MHB for colistin assays or 8ml of 5%LB-RPMI, for LL-37 assays. LL-37, colistin, or media control was added to a final concentration of 1mM and 0.5mg/L respectively. The cultures were shaken at 37°C for 1h and then spun at 3000 x *g* at room temperature for 5 min and re-suspended in 2ml of 10mM Tris buffer pH 8.0. The concentrated 2ml cultures were used to prepare 4ml bacterial stocks at OD_{600}=0.40 in 10mM Tris. Assays were conducted in a final volume of 200μl in triplicate in 96-well round bottom plates (Costar). Four conditions were tested. 1) 100μl of bacterial stock + 50μl of NPN (40μM final) + 50μl of 10 mM Tris. 2) 100μl of bacterial stock + 50μl of NPN + 50μl of EDTA (10 μM final). 3) 100μl of bacterial stock + 50μl of EDTA + 50μl of 10mM Tris. 4) 100μl of 10mM Tris + 50ml of NPN + 50ml of EDTA. As soon as all of the components were added and mixed, plates were immediately read in a fluorescent plate reader: excitation 250nm/emission 420nm. The NPN fluorescence signal from conditions 3) and 4), background, were subtracted from the signals measured from conditions 1) and 2). The NPN intensity from condition 1) bacteria+NPN was divided by the NPN signal measured from condition 2) bacteria+NPN+EDTA to obtain the percentage of permeability recorded in the presence of 10mM EDTA that permeabilizes the outer membrane of Gram-negative bacteria.

A. baumannii **foreign body infection model**. The *AB* foreign body infection studies were performed as previously described {Kadurugamuwa, 2003 #37}. Briefly, 14-gauge sterile non-pyrogenic intravenous catheters (Excel International) were cut into 1cm segments. These segments were then cut in half, so that the interior lumen was opened. Catheter fragments were sterilized by shaking in 70% ethanol for 2h and then rinsed twice in fresh LB. Catheter fragments were added to a 25ml starting culture of AB5075 in LB and grown overnight at 37°C with shaking. 8-week-old female CD1 mice (Charles River Labs) had the hair on their backs shaved and then removed with Nair (Naircare). The mice were anesthetized in an isoflurane chamber, their nude backs were sprayed with 70% ethanol, and a small 1cm incision was made. Tweezers were gently inserted into the incision site and used to open up a small subcutaneous pouch. A catheter fragment was removed from the overnight culture of MDR-*AB* and inserted into the pouch. The wound site was closed with Tegaderm film (3M) and the mice were placed on a

warm heating pad to recover. At 1, 24, and 48h after infection, the mice received a subcutaneous dose of 50mg/kg AZM, 100 mg/kg AZM, or 100μl PBS control. At 72h the mice were sacrificed with CO_2. The catheter fragment and all surrounding infected soft tissue was removed and placed in a 2 ml sterile micro tube (Sarstedt) containing 1ml of PBS and 1mm silica beads (Biospec) and the tissue was homogenized as detailed above for the lungs. Aliquots from each tube were serially diluted for CFU enumeration on LB plates. Three catheter segments per experimental run were removed from the overnight *AB* culture and homogenized in 1ml of PBS to determine the initial inoculum.

NBD-tagged AZM. 9a-NBD-AZM was synthesized as previously described {Matijasic, 2012 #30} with slight modifications made in the final step of the synthesis. To a solution of 9a-(3-aminopropyl)-9-deoxo-9a-aza-9a-homoerythromycin A (100mg, 0.126mmol) in dry EtOH (2ml) was added 4-chloro-7-nitrobenofurazan (25mg, 0.126mmol) and the resulting reaction mixture was stirred at room temperature for 5h. Water (5ml) was added to the reaction mixture and extracted with CH_2Cl_2 (3 x 5ml). The organic layers were combined, dried over Na_2SO_4 and concentrated under reduced pressure. Flash chromatography (CH_2Cl_2 to 10:1 CH_2Cl_2/MeOH) afforded the desired compound as a yellow solid (66 mg, 55.1%). NBD-tagged AZM: ^{1}H NMR (CD_3OD, 500MHz) δ 8.51 (d, J = 8.8 Hz, 1H), 6.42 (d, J = 9.0 Hz, 1H) –only the diagnostic benzofurazan shifts were noted (**Fig. S6A**); ^{13}C NMR (CD_3OD, 125 MHz) δ 177.7, 137.3, 102.1, 98.7, 95.6, 83.4, 79.0, 77.7, 77.1, 74.8, 74.5, 74.2, 73.0, 70.5, 67.2, 65.2, 64.5, 48.5, 44.9, 41.7, 40.9, 40.6, 38.8, 34.6, 34.3, 31.7, 31.4, 30.1, 29.4, 28.8, 28.2, 26.2, 24.8, 22.7, 22.3, 21.6, 21.1, 20.4, 20.2, 17.6, 16.4, 14.5, 13.1, 10.1, 8.8, 7.6; HRMS *m/z* calculated for $[C_{46}H_{79}N_6O_{15}]^+$: 955.5598, found 955.5597 (**Fig. S6B**).

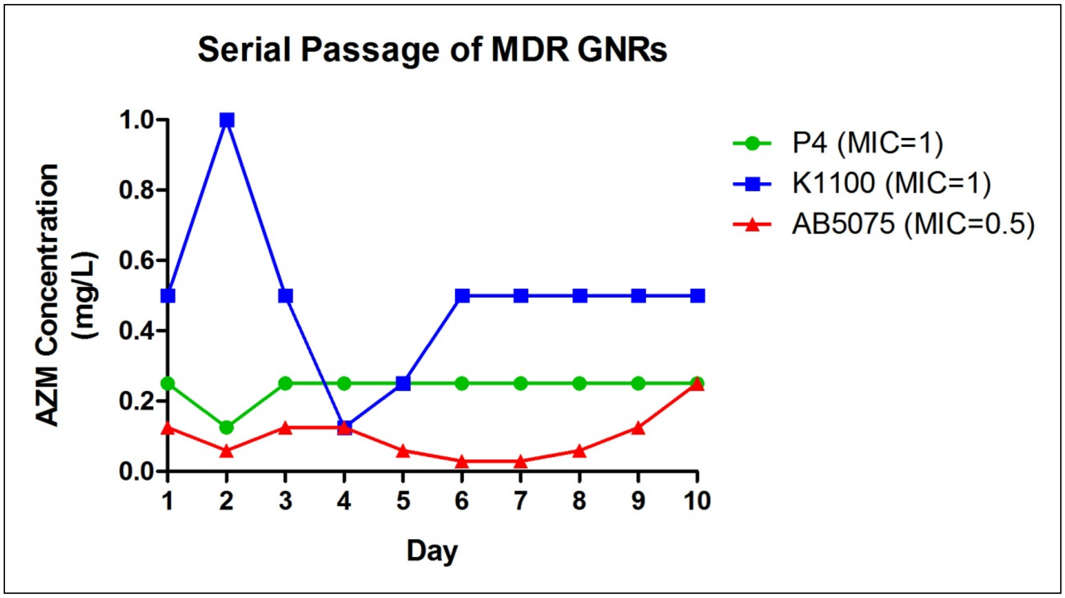

Day	*Pseudomonas aeruginosa*, Strain P4 (MDR) AZM (mg/L)	*Klebsiella pneumoniae*, Strain K1100 (MDR, KPC) AZM (mg/L)	*Acinetobacter baumannii*, Strain AB5075 (MDR) AZM (mg/L)
**1**	0.25	0.5	0.125
**2**	0.125	1	0.06
**3**	0.25	0.5	0.125
**4**	0.25	0.125	0.125
**5**	0.25	0.25	0.06
**6**	0.25	0.5	0.03
**7**	0.25	0.5	0.03
**8**	0.25	0.5	0.06
**9**	0.25	0.5	0.125
**10**	0.25	0.5	0.25

Abbreviations: AZM = Azithromycin; MDR = multidrug-resistant; KPC = *Klebsiella pneumoniae*, carbapenemase-producing; RPMI = Roswell Park Memorial Institute 1640, a basal mammalian tissue culture medium; LB = Luria broth.

Fig S1. Serial passage for 10 days of MDR GNR isolates at sub-minimum inhibitory concentrations of azithromycin to test for resistance evolution. On Day 0, bacteria were grown at 1/2 and 1/4 of their AZM MIC in 5ml of liquid culture - RPMI + 5%LB. Then 24 h after shaking at 37^0C, the highest concentration of AZM in which dense bacterial growth was observed was recorded. 50µl of this dense culture growth was then used to inoculate 3 new tubes containing 5ml of fresh media and 2, 1, and 1/2 the original concentration of AZM respectively.

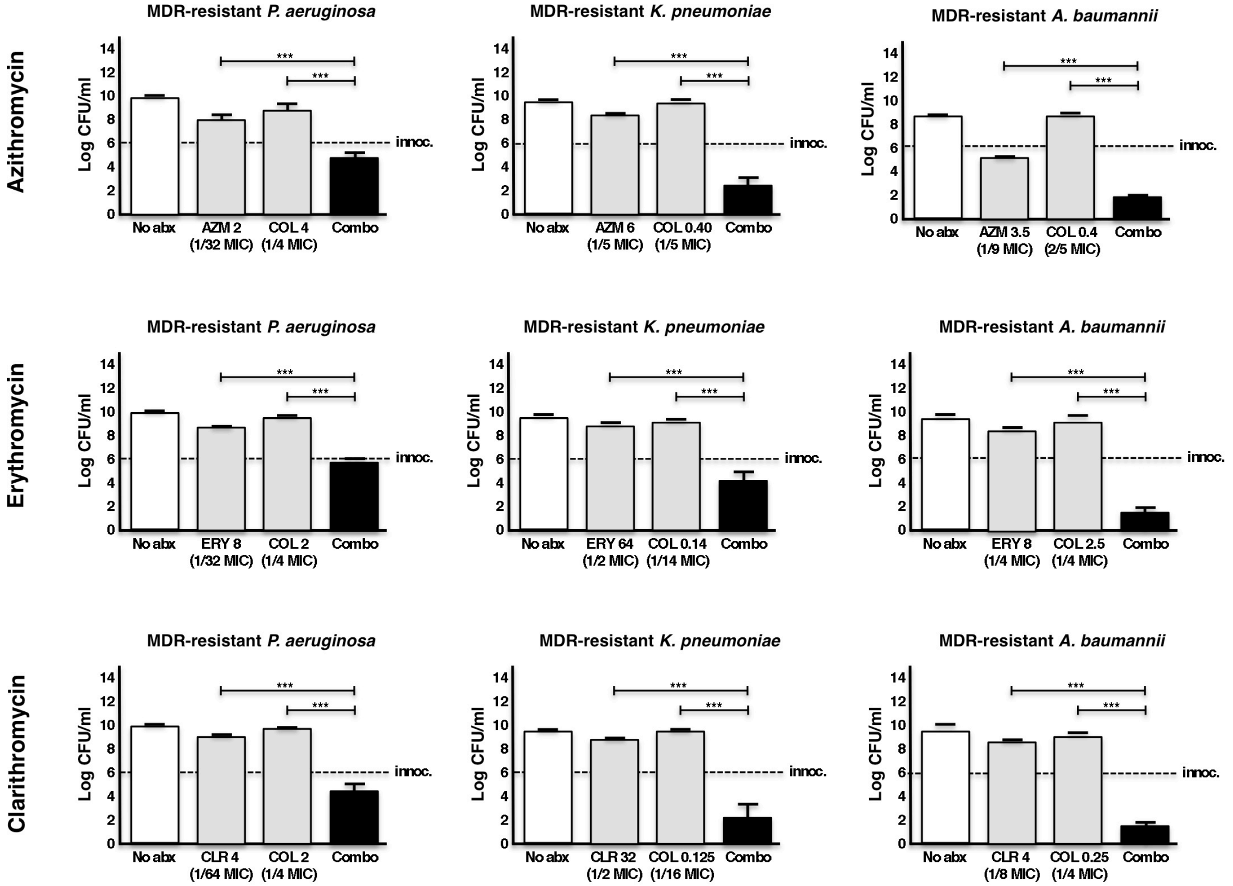

Fig. S2. Colistin synergizes with azithromycin, erythromycin, and clarithromycin to kill MDR GNR pathogens *in vitro*. All assays conducted using the standard bacteriologic media Ca-MHB. Time-kill assays demonstrate the effect of AZM, ERY, and CLR alone or in combination with colistin against MDR PA, KP and AB. Data plotted are mean ± SEM and represent the average of triplicates from two independent experiments. The starting bacterial innoculum ("innoc") is denoted by the dotted line. ***$P < 0.001$; one-way ANOVA.

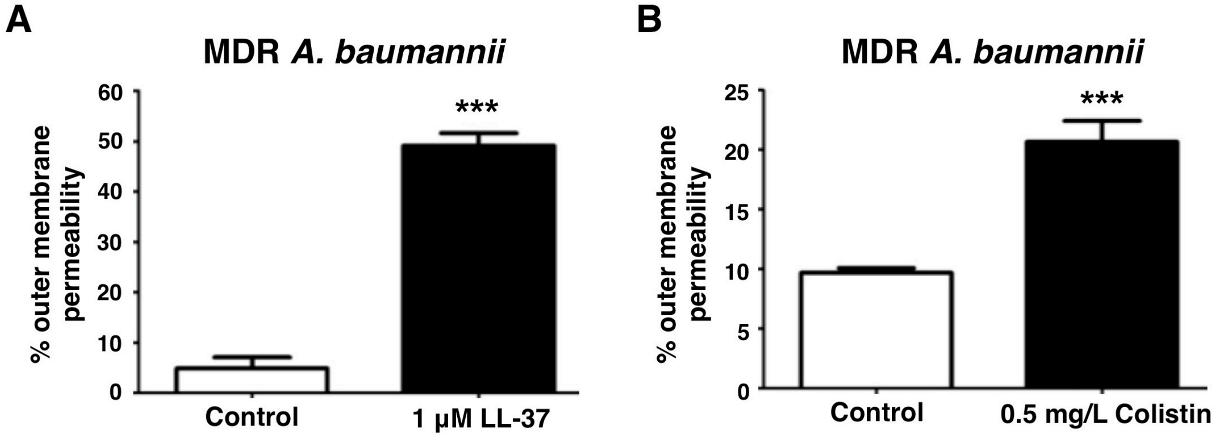

Fig. S3. Treating MDR *A. baumannii* with sub-MIC LL-37 or colistin significantly increases outer membrane permeability to 1-N-phenylnaphthylamine (NPN). Logarithmic growth phase MDR *AB* were treated for 1h with 1 μM LL-37 in RPMI+5%LB (**A**) or for 1h with 0.5 mg/L colistin in Ca-MHB (**B**). Bacteria were then washed with 10 mM Tris buffer and treated with 40 μM NPN. Data are expressed as % of the maximal value recorded in the presence of 10mM EDTA and represent the average of triplicates from 3 independent experiments. ***P<0.001; two-tailed student's t-test.

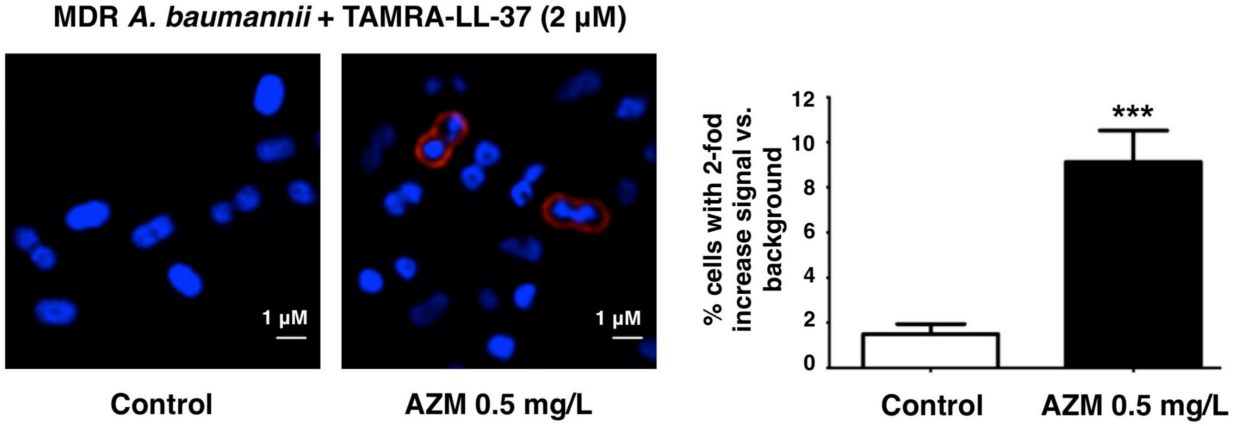

Fig. S4. Pre-treating MDR *A. baumannii* with AZM increases outer membrane binding of LL-37. Logarithmic growth phase MDR *AB* in RPMI+5%LB were incubated for 2h + 0.5 mg/L AZM. Bacteria were then treated for 30min with TAMRA-tagged LL-37 (red), washed, and then stained with DAPI (blue DNA stain). The number of MDR *AB* with red membrane signals at least twice the background level were counted using unbiased software analysis of multiple random microscopy fields with >500 cells counted per experimental replicate. Data plotted as the mean ± SEM and represent the combination of 3 independent experiments. ***P<0.001; two-tailed student's t-test.

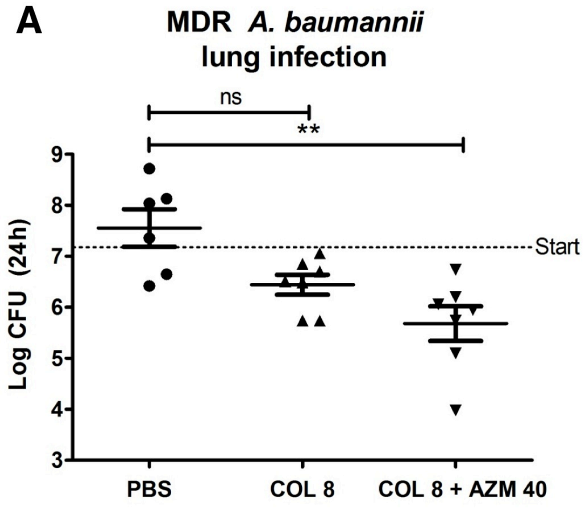

Fig. S5. Addition of low dose AZM to reduced dose colistin in treatment of MDR GNRs *in vivo*. (A) 8wk old female C57BL/6J mice were infected intratracheally (i.t.) with MDR *A. baumannii* strain AB5075. Lungs were homogenized at 24h; n = 6 for PBS control, 7 for colistin 8mg/kg, and 7 for AZM 40mg/kg ice. (B) 8wk old female C57BL/6J mice were infected intratracheally (i.t.) with MDR *AB*. Lungs homogenized at 24h; n = 7 for PBS control, 8 for colistin 6.5mg/kg, and 7 for AZM 32mg/kg ice. Data plotted as mean ± S.E.M. AZM was dosed once subcutaneously immediately after bacterial inoculation. Colistin was dosed once subcutaneously 1 h after the bacterial inoculation. Dashed line "start" denotes the initial inoculum. *P<0.05 and **P<0.01 based on one-way ANOVA analysis.

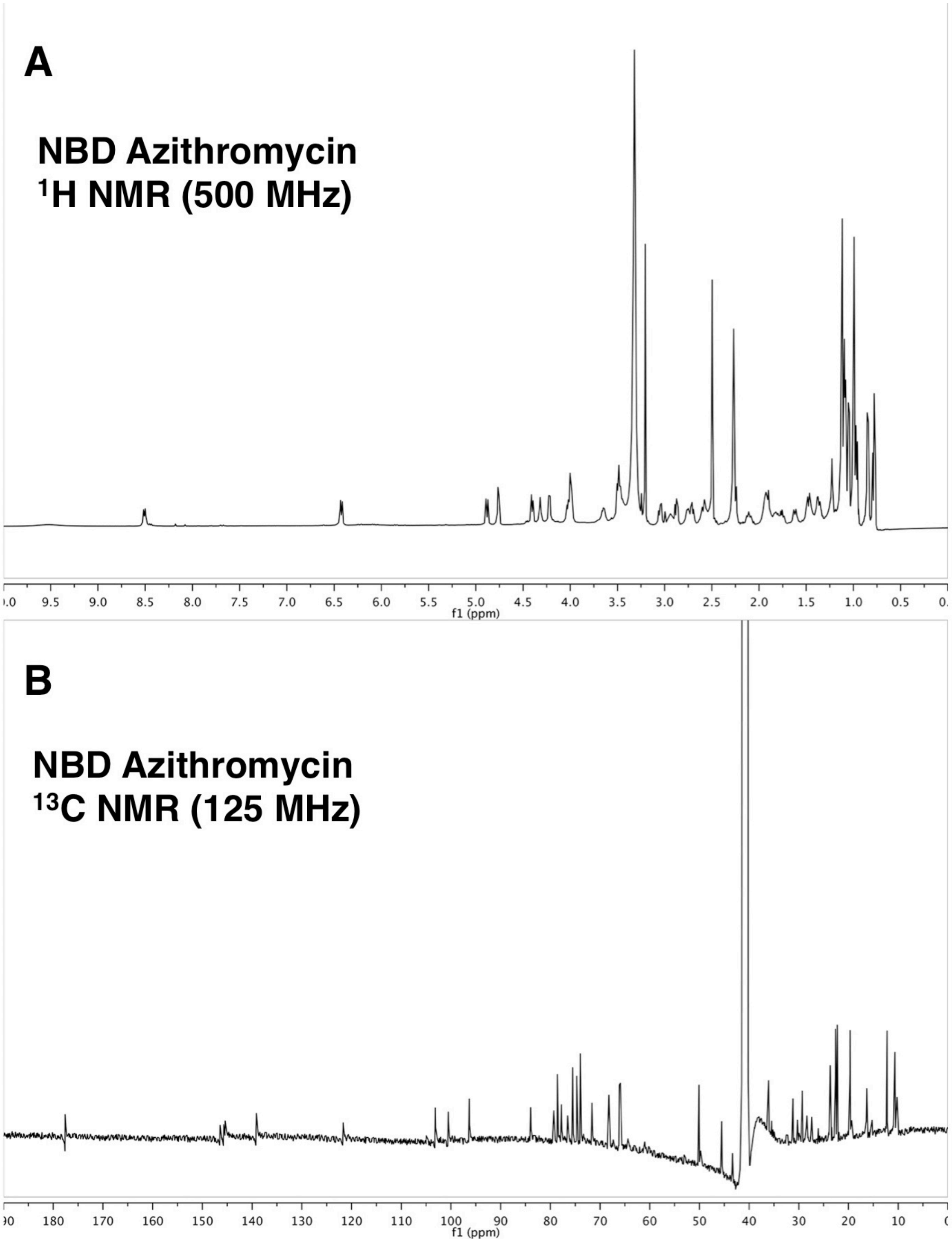

Fig. S6. Confirmation of structure and purity of synthesized NBD-tagged AZM. Shown are the spectra of (**A**) 1H NMR (500 MHz) and (**B**) 13C NMR (125 MHz) of NBD- tagged AZM in CD3OD.

ANTIBIOTIC	*Pseudomonas aeruginosa*, P4 (MDR)		*Klebsiella pneumoniae*, K1100 (MDR, KPC)		*Acinetobacter baumannii*, AB5075 (MDR)	
	MIC	Interpretation	MIC	Interpretation	MIC	Interpretation
Ampicillin	≥ 32	R	≥ 32	R	≥ 32	R
Amoxicillin/Clavulanate	≥ 32	R	≥ 32	R	≥ 32	R
Ampicillin/Sulbactam	≥ 32	R	≥ 32	R	≥ 32	R
Ticarcillin			≥ 128	R	≥ 128	R
Ticarcillin/Clavulanate	≥ 128	R				
Pipericillin	≥ 128	R	≥ 128	R	≥ 128	R
Pipericillin/Tazobactam	≥ 128	R	≥ 128	R	≥ 128	R
Cefalotin	≥ 64	R	≥ 64	R	≥ 64	R
Cefazolin	≥ 64	R	≥ 64	R	≥ 64	R
Cefuroxime	≥ 64	R	≥ 64	R	≥ 64	R
Cefuroxime Axetil	≥ 64	R	≥ 64	R	≥ 64	R
Cefotetan	≥ 64	R	8	R	≥ 64	R
Cefoxitin	≥ 64	R	32	R	≥ 64	R
Cefpodoxime	≥ 8	R	≥ 8	R	≥ 8	R
Cefotaxime	≥ 64	R	8	R	≥ 64	R
Ceftazidime	≥ 64	R	≥ 64	R	≥ 64	R
Ceftizoxime	≥ 64	R	4	R	≥ 64	R
Ceftriaxone	≥ 64	R	≥ 64	R	≥ 64	R
Cefepime	≥ 64	R	4	R	≥ 64	R
Aztreonam	≥ 64	R	≥ 64	R	≥ 64	R
Doripenem	≥ 8	R	≥ 8	R	≥ 8	
Ertapenem			≥ 8	R		
Imipenem	≥ 16	R	8	R	≥ 16	R
Meropenem	≥ 16	R	≥ 16	R	≥ 16	R
Amikacin	32	I	≥ 64	R	≥ 64	R
Gentamicin	8	I	≥ 16	R	≥ 16	R
Tobramycin	≤ 1	S	≥ 16	R	8	I
Nalidixic Acid	≥ 32	R	≥ 32	R	≥ 32	R
Ciprofloxacin	≥ 4	R	≥ 4	R	≥ 4	R
Levofloxacin	≥ 8	R	≥ 8	R	4	I
Moxifloxacin	≥ 8	R	≥ 8	R	≥ 8	R
Norfloxacin	8	I	≥ 16	R	≥ 16	R
Tetracycline	≥ 16	R	4	S	≤ 1	S
Tigecycline	≥ 8	R	4	I	≤ 0.5	S
Nitrofurantoin	≥ 512	R	128	R	≥ 512	R
TMP/SFX	≥ 320	R	40	S	≥ 320	R

Abbreviations: MDR = Multidrug-resistant; KPC = *Klebsiella pneumonia,* carbapenemase-producing; R = Resistant; I = Intermediate; S = Sensitive; TMP/SFX = Trimethoprim/Sulfamethoxazole

Table S1. Three multidrug (including carbapenem)-resistant Gram-negative bacterial strains examined in this study. Automatic minimum inhibitory concentration (MIC) testing results in cation-adjusted Mueller-Hinton broth by VITEK - 2 testing system.

Bacterial Species/Strain	**Azithromycin** MIC (mg/L)		**Ciprofloxacin** MIC (mg/L)		**Erythromycin** MIC (mg/L)		**Clarithromycin** MIC (mg/L)		**Colistin** MIC (mg/L)		LL-37 MIC (μM)
	Ca-MHB	RPMI + 5% LB	Ca-MHB	RPMI + 5% LB	Ca-MHB	RPMI + 5% LB	Ca-MHB	RPMI + 5% LB	Ca-MHB	RPMI + 5% LB	RPMI + 5% LB
Pseudomonas aeruginosa, Strain PA01	> 64	2	0.8	0.8							
Pseudomonas aeruginosa, Strain P4 (MDR)	> 64	1	12	12	>256	8	>256	8	8	4	64
Klebsiella pneumoniae, Strain K700603	64	2	< 0.5	< 0.5							
Klebsiella pneumoniae, Strain K1100 (MDR, KPC)	32	1	> 25	> 25	128	4	64	4	2	2	32
Acinetobacter baumannii, Strain AB19606	64	0.25	2	3							
Acinetobacter baumannii, Strain AB5075 (MDR)	32	0.5	> 25	> 25	32	1	32	0.5	1	2	4

Abbreviations: Ca-MHB = Cation-adjusted Mueller-Hinton broth; RPMI = Roswell Park Memorial Institute 1640, a basal mammalian tissue culture medium; LB = Luria broth; MDR = multidrug-resistant; KPC = *Klebsiella pneumonia,* carbapenemase-producing.

Table S2. Minimum inhibitory concentration (MIC) of Gram-negative bacterial strains used in this study to azithromycin, ciprofloxacin, erythromycin, clarithromycin, colistin and human cathelicidin LL-37. Comparative testing performed in standard bacteriologic testing media or mammalian tissue culture medium.

**Bacterial Species/Culture Site**	**Azithromycin** MIC (mg/L)	
	Ca-MHB	RPMI + 5% LB
Pseudomonas aeruginosa, WP2 (MDR) - Sputum	32	1
Pseudomonas aeruginosa, WP3 (MDR) - Wound	256	0.5
Pseudomonas aeruginosa, WP5 (MDR) - Sputum	32	<0.06
Pseudomonas aeruginosa, UCSD P1 (MDR) - Urine	128	2
Klebsiella pneumoniae, WK7 (MDR) - Urine	256	32
Klebsiella pneumoniae, WK8 (MDR) - Urine	32	1
Klebsiella pneumoniae, WK9 (MDR) - Urine	32	0.5
Klebsiella pneumoniae, WK10 (MDR) - Sputum	>256	32
Acinetobacter baumannii, WA2 (MDR) - Wound	32	0.125
Acinetobacter baumannii, WA4 (MDR) - Peri-anal	64	0.125
Acinetobacter baumannii, WA5 (MDR) - Sputum	64	0.125

Abbreviations: Ca-MHB = Cation-adjusted Mueller-Hinton broth; RPMI = Roswell Park Memorial Institute 1640, a basal mammalian tissue culture medium; LB = Luria broth; MDR = multidrug-resistant.

Table S3. Minimum inhibitory concentration (MIC) of azithromycin vs. a panel of contemporary clinical isolates of multidrug-resistant Gram-negative bacteria. Comparative testing performed in standard bacteriologic testing media or mammalian tissue culture medium.
